# An observational study of the efficacy of combined orthodontic–systemic periodontal treatment

**DOI:** 10.1097/MD.0000000000043373

**Published:** 2025-07-11

**Authors:** Shiwei Miao, Yinghong Wu, Hong Ning, Junjie Xu

**Affiliations:** a Department of Stomatology, Nantong Third People’s Hospital, Affiliated Nantong Hospital 3 of Nantong University, Nantong, Jiangsu, China.

**Keywords:** interleukin-1β, interleukin-6, orthodontics, periodontitis, systemic periodontal treatment, tumor necrosis factor-α

## Abstract

This study comparatively analyzes the periodontal status of patients at 6 months after combined orthodontic–systemic periodontal treatment for improving clinical treatment protocols. Eighty periodontitis patients treated in our hospital from June 2019 to June 2022 were selected as subjects and randomly divided into observation and control groups, with 40 patients in each group. The observation group underwent combined orthodontic–systemic periodontal treatment; the control group underwent systemic periodontal treatment. All subjects had been followed up for 6 months, they underwent periodontal clinical examination and gingival crevicular fluid detection at 6 months after treatment; the effective rate was compared between 2 groups. The total effective rate was higher in the observation group (97.50%) than in the control group (87.50%). Compared with before treatment, the clinical attachment loss, gingival bleeding index, periodontal probing depth, plaque index, bleeding on probing, tumor necrosis factor-α, interleukin-6, and interleukin-1β levels in gingival sulcus fluid were significantly decreased in 2 groups at 6 months after treatment, which were lower in the observation group than in the control group, with statistically significant differences (*P* < .05). Combined orthodontic–systemic periodontal treatment can increase the therapeutic effect, improve periodontal status and enhance anti-inflammatory response in periodontitis patients, thus promoting their recovery.

## 1. Introduction

As one of the most important oral diseases that contribute to the global burden of chronic diseases, periodontitis is a major public health problem, and it is a chronic inflammation mainly caused by bacteria located in dental plaque, which can cause a significant damage to periodontal supporting tissues such as gingiva and periodontal membrane.^[1]^ So far, many studies have confirmed that periodontal health has a close correlation with the occlusal trauma and occlusal disorders.^[2,3]^ At present, periodontal tissue regeneration is the most effective intervention for the treatment of periodontitis, which can enable the periodontal membrane to grow and form new periodontal tissue, with a significant therapeutic effect.^[4]^ However, some studies have shown that a simple periodontal sequence treatment cannot meet the requirements of patients for tooth beauty,^[5]^ and various coping strategies have been widely studied, including targeted small molecule drugs and hydrogels, etc.^[6,7]^ Orthodontic treatment can solve the problem of tooth deformity by rearranging the teeth, improving the chewing ability and ensure the tooth beauty at the same time, which may increase the benefits for patients.^[8]^ The inflammatory cytokines, as important inflammatory indexes in gingival crevicular fluid, have important guiding significance for the evaluation of the treatment and control effect of periodontitis.^[9]^ Based on this, a total of 80 patients with periodontitis were selectively enrolled into this prospective cohort study. The effects of combined orthodontic–systemic periodontal treatment on the visual analog scale (VAS) score, and tumor necrosis factor-α (TNF-α), interleukin-6 (IL-6), and interleukin-1β (IL-1β) levels in gingival sulcus fluid of periodontitis patients were assessed, so as to determine whether the combined orthodontic–systemic periodontal treatment can improve the objective and subjective treatment benefits of patients, now it is reported as follows.

## 2. Materials and methods

### 2.1. Study subjects

From June 2019 to June 2022, a total of 40 patients with periodontitis who received combined orthodontic–systemic periodontal treatment were selected as the observation group, and 40 age- and gender-matched patients with periodontitis who received systemic periodontal treatment alone at the same time were selected as the control group. Inclusion criteria: (1) patients with periodontitis who met the relevant diagnostic criteria of periodontitis in the Guidelines for The Diagnosis and Treatment of Periodontal Diseases and were definitely diagnosed by imaging examinations such as X-ray^[10]^; (2) those who did not receive other periodontal treatments at 3 months before enrollment; and (3) those who cooperated with us during follow-up visit. Exclusion criteria: (1) patients with oral malignant diseases; (2) those with abnormal coagulation function; (3) pregnant or lactating women; and (4) those with other malignant tumors, abnormal hepatorenal function, abnormal basic metabolism, serious autoimmune diseases, and serious infectious diseases. All patients were required to sign an informed consent form, and this study was approved by the medical ethics committee of our hospital. General data such as gender, age and course of disease were collected from all patients in the 2 groups, and the initial course data such as the type of malocclusion deformity and severity of periodontitis of the patients were determined through orthodontic and periodontal examinations.

### 2.2. Treatment method

At first, the patients’ periodontal status was comprehensively evaluated, the indexes such as periodontal probing depth (PPD), clinical attachment loss (CAL), plaque index (PI), gingival bleeding index (GBI) and bleeding on probing (BOP) were detected, and the occlusal status of patients was evaluated according to Angle classification of malocclusion classification. The basic periodontal treatment included supragingival scaling and subgingival curettage. During the acute inflammatory period, some drugs such as amoxicillin and metronidazole could be used to control inflammation before periodontal surgery. The patients in the control group were treated with periodontal tissue regeneration: after the gingiva was washed, the suppurative and inflammatory tissues were scraped off, and then root planning was performed; full-scale curettage was performed in patients with deep periodontal pockets, and a periodontal flap surgery should be carried out if necessary. An artificial bone was placed in the area of periodontal bone defect, the mucoperiosteal flap was sutured, and the damaged gingival tissue was removed. On the basis of the above treatment, the patients in the observation group underwent orthodontic treatment: an adhesive molar forward-pushing buccal tube was used for fixation according to the situation; a vertical straight wire appliance was placed in the area of the displaced tooth, and the tooth was further straightened and aligned using titanium nickel wire; the tooth space was closed to 2–3 mm, the fixed orthodontic appliance was used to bond the bracket; the arch wire was bent and adjusted to complete the sequence orthodontic treatment, and the patient should pay attention to oral hygiene and regular reexamination during treatment. All patients in the 2 groups were followed up for at least 6 months after surgery, during which oral health education was conducted to guide the patients to perform correct oral cleaning.

### 2.3. Observational index

#### 2.3.1. Clinical efficacy

The clinical efficacy was divided into cured, effective and ineffective, of which, “cured” means that after treatment, all clinical symptoms such as periodontal pain, redness and swelling disappear, and the periodontal tissue gradually returned to normal; “effective” means that after treatment, the clinical symptoms such as periodontal pain, redness and swelling were reduced, and the periodontal tissue was improved; “ineffective” means that the clinical symptoms such as periodontal pain, redness and swelling were not obviously reduced, as well as the periodontal tissue had not been significantly improved. The total effective rate is equal to the curative rate plus the effective rate. The pain situation was evaluated and reported by the patients themselves using the VAS, where 0 represents no pain and 10 represents severe pain; the patients were followed up respectively before surgery and at 7 days, 3 and 6 months after surgery.

#### 2.3.2. Periodontal status

The information such as CAL, GBI, PPD, PI, and BOP of patients in the 2 groups before treatment and at 3 and 6 months after treatment were recorded and analyzed by visual observation and probing. PPD: the distance between the gingival margin and the bottom of the gingival pocket or between the gingival margin and the bottom of the gingival sulcus was measured using a periodontal probe, and mm was selected as the measurement unit; CAL: after the depth of the periodontal probe was measured, the cemento-enamel junction was explored and its distance from the gingival margin was measured as the tip of the periodontal probe withdrew along the root surface of the tooth; PI: the periodontal probe was used to gently scratch the tooth surface, and scoring was performed according to the thickness and area of the plaque, with a score range of 0 to 3 points, and a lower score indicated a better periodontal cleaning status in the patient. GBI: the gingival bleeding tendency was observed, with a score range of 0 to 3 points, and a lower score indicated less gingival bleeding. BOP: a periodontal probe with a scale in its blunt head was used for probing, and the probe was parallel to the long axis of the tooth, and the probe tip was attached to the tooth surface; excessive force should not be exerted during probing, and the fulcrum should be fixed; the bleeding status was recorded after probing.

#### 2.3.3. Inflammatory factor level

Two milliliters of gingival crevicular fluid was collected from each of patients in the 2 groups before treatment and at 6 months after treatment, and the TNF-α, IL-6 and IL-1β levels in gingival crevicular fluid were detected by enzyme-linked immunosorbent assay.

#### 2.3.4. Statistical methods

SPSS 25.0 statistical software was used for data analysis, and chi square test was conducted for counting data (total effective rate); the measurement data (CAL, GBI, PPD, PI, BOP, and inflammatory factor level in the gingival sulcus fluid) were expressed as the mean ± standard deviation, and *t* test was performed. *P* < .05 indicated that the difference was statistically significant between 2 groups.

## 3. Results

### 3.1. General information

A total of 80 patients were included in this study, there were 22 males and 18 females among 40 patients in the control group, with an average age of 46.32 ± 2.96 years (24–62 years). There were 26 males and 14 females among 40 patients in the observation group, with an average age of 47.92 ± 3.52 years (23–60 years). The course of disease before treatment in periodontitis patients of the 2 groups was between 1 year and 4 years. The analysis of the disease course and the intergroup comparison showed that there was no significant difference in the general data between 2 groups (*P* > .05), indicating the comparability between the groups (Table 1). The evaluation and statistical analysis of the type of malocclusion deformity showed that there was no significant difference in the distribution of the type of malocclusion deformity between the 2 groups (*P* > .05) (Table 2).

**Table 1:** Comparison of general data between 2 groups.

	Control group	Observation group	*P* value
Sex ratio	22:18	26:14	.593
Average age (yr)	46.32 ± 2.96	47.92 ± 3.52	.792
Course of disease (yr)	1–4	1–4	.688

**Table 2:** Comparison of the type of malocclusion deformity between 2 groups.

	Control group	Observation group	*P* value
Angle I	8	6	.766
Angle II	21	19	.892
Angle III	11	15	.539

### 3.2. Clinical efficacy

**Figure 1.**
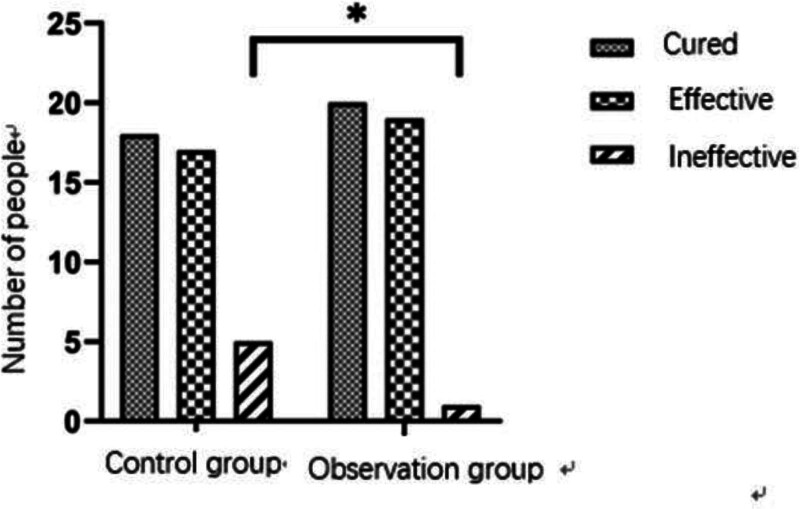
Comparison of the total clinical effective rate between the observation group and the control group. The observation group demonstrated a significantly higher effective rate compared to the control group (*P* < .05).

**Figure 2.**
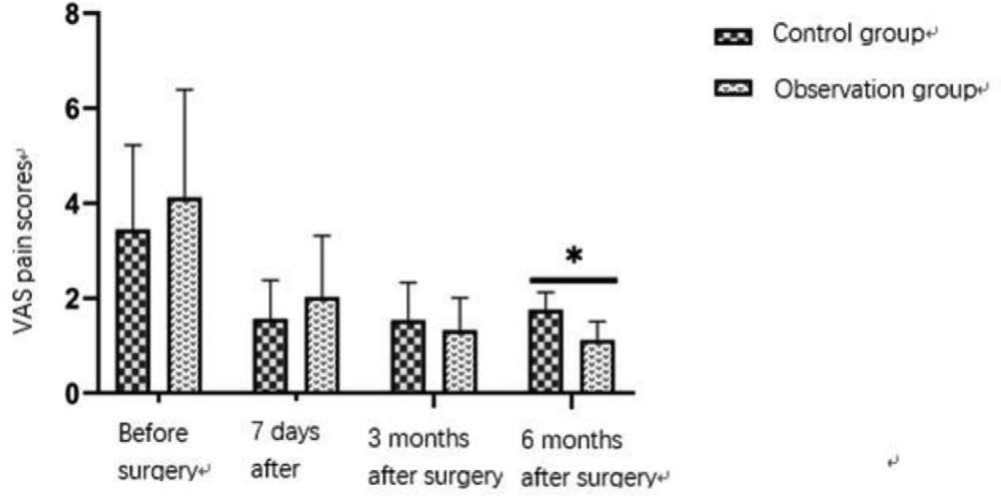
Visual analog scale (VAS) pain scores of the observation group and control group at different time points. There was no significant difference in pain scores before surgery and 3 months postsurgery. However, at 6 months postsurgery, the observation group showed significantly lower pain scores compared to the control group (*P* < .05).

Among the 80 patients included in this study, the total clinical effective rate of 40 patients in the observation group was higher than that in the control group, and there was a statistical difference between the 2 groups (*P* < .05) (Table 3 and Fig. 1). During the follow-up, VAS scores showed that there was no significant difference in subjective pain score between the 2 groups before and at 3 months after the surgery, but the subjective pain scores of patients in the observation group were better than those in the control group at 6 months after the surgery (*P* < .05) (Table 4 and Fig. 2).

**Table 3:** Comparative evaluation of clinical efficacy at 6 months between 2 groups.

Group	Cured	Effective	Ineffective	Total effective rate
Control group	18	17	5	87.5%
Observation group	20	19	1	97.5%
*P* value				.032*

* *P*-value of <.05 indicated a statistically significant difference.

**Table 4:** Comparison of VAS pain scores between 2 groups.

Group	Before surgery	7 days after surgery	3 months after surgery	6 months after surgery
Control group	3.45 ± 1.77	1.57 ± 0.81	1.54 ± 0.79	1.76 ± 0.36
Observation group	4.12 ± 2.27	2.03 ± 1.29	1.33 ± 0.68	1.12 ± 0.39
*P* value	.596	.577	.639	.042*

VAS = visual analog scale.

* *P*-value of <.05 indicated a statistically significant difference.

### 3.3. Periodontal status

**Figure 3.**
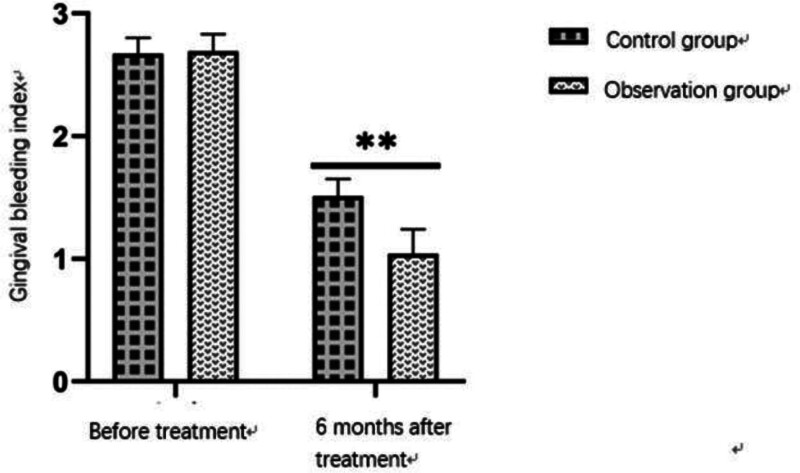
Changes in gingival bleeding index (GBI) before and 6 months after treatment in the observation and control groups. Both groups exhibited significant improvement, with the observation group showing significantly better outcomes than the control group (*P* < .01).

**Figure 4.**
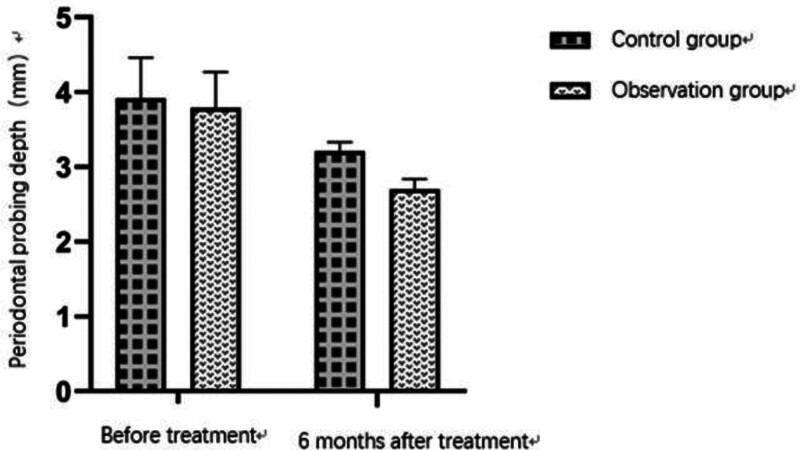
Changes in periodontal probing depth (PPD) before and 6 months after treatment in the observation and control groups. Both groups showed significant reductions in PPD, with greater improvement observed in the observation group (*P* < .05).

The follow-up at 6 months after treatment showed that the periodontal statuses in the 2 groups were significantly improved compared with those before surgery (*P* < .05). The CAL, GBI, PPD, and PI of patients were significantly reduced compared with those before surgery. The BOP in the 2 groups was significantly improved. The BOP rate in the observation group at 6 months after surgery was 12.5%, which was significantly lower than 27.5% in the control group. Further intergroup comparison showed that the recovery of patients in the observation group was better than that in the control group, with a statistically significant difference between 2 groups (*P* < .05). See Tables 5, 6 and Figures 3 and 4 for details.

**Table 5:** Comparison of periodontal status between 2 groups.

Group	CAL (mm)		GBI		PPD (mm)	
	Before treatment	6 months after treatment	Before treatment	6 months after treatment	Before treatment	6 months after treatment
Control group	3.18 ± 0.32	1.19 ± 0.23	2.67 ± 0.13	1.51 ± 0.14	3.92 ± 0.54	3.21 ± 0.12
Observation group	3.11 ± 0.49	1.01 ± 0.18	2.69 ± 0.14	1.04 ± 0.20	3.79 ± 0.48	2.70 ± 0.14
*P* value	.873	.049*	.692	.009*	.133	.012*

CAL = clinical attachment loss, GBI = gingival bleeding index, PPD = periodontal probing depth.

* *P*-value of <.05 indicated a statistically significant difference.

**Table 6:** Comparison of periodontal status between 2 group.

Group	PI		BOP (case, %)	
	Before treatment	After treatment	Before treatment	After treatment
Control group	2.29 ± 1.12	1.38 ± 0.33	40 (100%)	11 (27.5%)
Observation group	2.11 ± 1.09	0.91 ± 0.15	40 (100%)	5 (12.5%)
*P* value	.692	.028*	–	.009*

PI = plaque index, BOP = bleeding on probing.

* *P*-value of <.05 indicated a statistically significant difference.

### 3.4. Inflammatory factor level

The ELISA test showed that the levels of inflammatory factors such as TNF-α, IL-1β, and IL-6 in gingival crevicular fluid in patients of the 2 groups after systematic periodontal treatment were lower than those before treatment, and the levels of inflammatory factors in the observation group were lower than those in the control group. After treatment, there were statistically significant differences in the expressions of inflammatory factors between the 2 groups (all *P* < .05), as shown in Table 7.

**Table 7:** Comparison of inflammatory factors in gingival crevicular fluid between 2 group.

Group	TNFα (mg/L)		IL-1β (mg/L)		IL-6 (mg/L)	
	Before treatment	After treatment	Before treatment	After treatment	Before treatment	After treatment
Control group	3.58 ± 0.39	1.43 ± 0.32	77.84 ± 8.01	61.86 ± 7.98	8.75 ± 1.39	5.83 ± 0.68
Observation group	3.63 ± 0.27	1.04 ± 0.29	78.64 ± 8.82	55.73 ± 7.63	8.32 ± 0.98	3.21 ± 0.76
*P* value	.879	.027*	.832	.013*	.533	.009*

* *P*-value of <.05 indicated a statistically significant difference.

## 4. Discussion

Periodontitis is a chronic inflammation that occurs in periodontal tissue. The main function of the periodontal tissue is to support, fix and nourish the teeth, and its components include alveolar bone, gingiva, periodontal membrane and cementum. With the development of the disease, inflammation can affect the gingival soft tissue and the supporting tissue deep in the teeth, and the serious lesions may lead to tooth loosening or loss, which is seriously detrimental to oral health and is also one of the public health problems in the world today.^[10]^ The main predisposing factors of periodontitis include bacterial infection (mainly caused by porphyromonas gingivitis), occlusion disorder and occlusion trauma.^[11,12]^ During the onset of periodontitis, the main symptoms are gingival redness, swelling, tenderness, easy bleeding, oral odor and changes in dental occlusion, which not only affect the beauty of teeth, but also reduce the chewing ability and chewing efficiency. Therefore, how to improve the therapeutic effect of periodontitis has become the focus of clinical attention. On the one hand, periodontal tissue regeneration and systematic periodontal treatment can create conditions for patients’ oral orthodontic treatment, thereby fundamentally eliminating the predisposing factors of periodontitis. Systematic periodontal treatment will not only repair the damaged periodontal tissue, but also promote the regeneration of alveolar bone and normal periodontal tissue in a short time. The common types of alveolar bone destruction in clinic are horizontal absorption and vertical absorption. Both horizontal and vertical absorption of alveolar ridge can affect the height of alveolar ridge, reduce the bone volume around the root and increase the tooth mobility to a certain extent. However, it is worth noting that simple periodontal treatment cannot correct the malocclusion deformity. Under the action of continuous disorder or overload stress, the teeth in the mouth may be displaced again and occlusal trauma may occur over time, thus the efficacy of periodontal maintenance treatment is reduced, which will become one of the risk factors inducing periodontitis. Therefore, combined orthodontic–systemic periodontal treatment can improve the therapeutic effect, effectively inhibit inflammatory response and improve the condition of patients.

At present, as the first choice to correct the malocclusion deformity, oral orthodontic treatment can better arrange the teeth, improve the patient’s occlusal relationship, facilitate the recovery of periodontal tissue, ensure the occlusal balance, and thus promote the recovery of the disease. Therefore, the joint intervention of orthodontic–systemic periodontal treatment has a significant effect on periodontitis. In the treatment process of periodontitis, adjusting the occlusal relationship and dentition position can improve the therapeutic effect of periodontal treatment, make it easy to maintain, and optimize the feasibility and operation mode of periodontal–orthodontic treatment, thus better meeting the clinical needs of periodontal treatment. Zuo Changyan et al^[11]^ found that the periodontal tissue regeneration combined with orthodontic treatment had better efficacy in periodontitis patients. The conclusions of our study had a good consistency with the above results, and our study further indicated that not only periodontal tissue regeneration, but also orthodontic-assisted sequence periodontal treatment was of great significance in controlling the periodontal environment and maintaining the stability of periodontal conditions.

In this study, the total clinical effective rate of patients in the observation group was higher than that in the control group; in addition, the subjective VAS scores of patients were improved after surgery compared with those before surgery, and there was a difference in subjective VAS score between the observation group and the control group at 6 months after surgery, which may indicate that the combined orthodontic–systemic periodontal treatment can be tolerated by patients after application of a low orthodontic force, and is conducive to the maintenance of periodontal environment. Clinical examination revealed that the CAL, GBI, PPD, PI, and PBR of the patients in the observation group were lower than those in the control group at 3 and 6 months after treatment, suggesting that combined orthodontic–systemic periodontal can improve the efficacy, periodontal status and disease condition in the treatment of periodontitis patients, and help maintain a long-term stability.

Relevant reports suggest that the development of periodontitis is related to humoral immunity. Therefore, 3 typical inflammatory factors such as TNF-α, IL-1β, and IL-6 TNF were mainly selected during the detection of inflammation-related factors in gingival crevicular fluid. Of which, TNF-α is one of the main cytokines participating in the inflammation-induced local alveolar bone resorption, it has regulatory effects on bone marrow mesenchymal stem cells, osteoblasts and osteoclasts, and may be a key factor for the periodontal disease to cause soft and hard tissue injury.^[12]^ The concentration of IL-1β is closely correlated with the severity of periodontitis. A low concentration of IL-1β can promote the expression of osteoprotegerin messenger RNA, while a high concentration of IL-1β can hinder the expression of osteoprotegerin messenger RNA, aggravate the inflammatory response, further damage the repair ability of periodontal tissue, and also have a negative effect on the migration and proliferation of gingival epidermal cells,^[13]^ which can indirectly regulate the differentiation and maturation of osteoclasts, resulting in the destruction of alveolar bone. During the development of periodontitis, as a multifunctional cytokine, IL-6 can hinder the growth of periodontal ligament cells, thereby weakening the repair and metabolism of periodontal tissue, inducing osteoblasts to produce osteoclast differentiation factors, transforming osteoclast precursor cells into osteoclasts, and causing degradation of bone matrix; at the same time, IL-6 can induce B lymphocytes to differentiate and secrete immunoglobulins, promote the proliferation of a variety of cells, and participate in bone resorption and periodontal tissue destruction.^[14,15]^ Systemic periodontal treatment can significantly alleviate inflammation, but there is no more precise and effective means for occlusal trauma caused by malocclusion deformity, plaque accumulation caused by poor dentition arrangement, etc, while the combined orthodontic–systemic periodontal treatment can minimize periodontal inflammation, fundamentally eliminate the predisposing factors and aggravating factors of such periodontitis, and help maintain long-term stability after periodontal treatment, which is similar to previous studies.^[8]^ In this study, the expression level of relevant inflammatory factors in gingival crevicular fluid of patients in the observation group was lower than that in the control group at 6 months after treatment, suggesting that oral orthodontics combined with periodontal tissue regeneration can have a good efficacy in the treatment of periodontitis patients, effectively inhibit inflammatory response, and promote the improvement of patients’ condition.

## 5. Conclusions

The use of combined orthodontic–systemic periodontal treatment can enhance the therapeutic effect, improve the periodontal status, inhibit the inflammatory response, and thus increase the benefits to patients with periodontitis, which is worthy of clinical application.

## Acknowledgments

The authors are grateful for the support of Nantong Livelihood Science and Technology Program Instructional Project of Nantong Science and Technology Bureau.

## Author contributions

**Conceptualization:** Yinghong Wu.

**Data curation:** Yinghong Wu, Hong Ning.

**Formal analysis:** Yinghong Wu, Hong Ning.

**Supervision:** Junjie Xu.

**Writing – original draft:** Shiwei Miao.

**Writing – review & editing:** Shiwei Miao.
